# Calcium supplementation during pregnancy and maternal and offspring bone health: a systematic review and meta‐analysis

**DOI:** 10.1111/nyas.14705

**Published:** 2021-11-15

**Authors:** Kati Tihtonen, Päivi Korhonen, Jaana Isojärvi, Riitta Ojala, Ulla Ashorn, Per Ashorn, Outi Tammela

**Affiliations:** ^1^ Department of Obstetrics and Gynecology Tampere University Hospital Tampere Finland; ^2^ Faculty of Medicine and Health Technology, Center for Child, Adolescent, and Maternal Health Research Tampere University Tampere Finland; ^3^ Department of Pediatrics Tampere University Hospital Tampere Finland; ^4^ Library Tampere University Tampere Finland

**Keywords:** pregnancy, calcium supplementation, vitamin D, offspring, bone mineral density

## Abstract

Insufficient calcium intake during pregnancy may lead to maternal bone resorption and lower bone density of offspring. We evaluated the impact of supplementary calcium with or without vitamin D during pregnancy on maternal and offspring bone mineral density (BMD) and teeth firmness of the offspring. Randomized controlled trials (RCTs) were searched systematically in 11 databases. Two researchers independently screened the titles and abstracts of 3555 records and the full texts of 31 records to examine eligibility. The search yielded seven RCTs (11 reports, *n* = 1566).  No advantage of calcium supplementation was found on maternal BMD after delivery or during breastfeeding, or on offspring BMD, even when dietary calcium intake was low. The results were neither modified by the dose of calcium nor concomitant vitamin D administration. A suspicion of some long‐term harm of the intervention on maternal BMD and growth of female offspring was raised based on the data. One study suggested some benefit of high‐dose calcium supplementation on offspring teeth firmness at 12 years old. A low number of the studies and abundant missing data reduced the quality of the findings. The impact of calcium supplementation on maternal and offspring bone health was deemed unknown because of inconclusive research results.

## Graphical abstract

Insufficient calcium intake during pregnancy may lead to maternal bone resorption and lower bone density of offspring. The aim of this systematic review was to synthesize the available data from randomized controlled trials to establish the impact of calcium supplementation, alone or with vitamin D during pregnancy, on maternal and offspring bone health and teeth firmness.

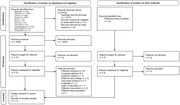

## Introduction

Pregnancy and breastfeeding could compromise maternal bones due to increased need of calcium to allow mineralization of the skeleton of the growing fetus and the infant. During pregnancy, the absorption of calcium from the intestine is increased, correlating directly with maternal calcium intake. Calcium is actively transported to the fetus by the placenta. If calcium intake is low, calcium release from the maternal bones becomes prevalent toward the end of the pregnancy.^1^ This may lead to low maternal bone mineral density (BMD), risk of delayed bone maturation of the newborn, and decreased BMD or teeth firmness of the offspring in later life.

Calcium intake is lower than recommended in large areas of the world.^2^ In such areas, supplementation is recommended during pregnancy to avoid preeclampsia.^3^ Theoretically, such supplementation might be expected to also positively affect maternal or child bone health. The aim of this systematic review was to synthesize the available data from randomized controlled trials (RCTs) to establish the impact of calcium supplementation, alone or with vitamin D during pregnancy, on maternal and offspring bone health and teeth firmness. The goal was also to investigate whether the impact of calcium is dependent on supplementary dose or maternal baseline dietary calcium intake.

## Methods

The study protocol was planned according to the Preferred Reporting Items For Systematic Review And Meta‐Analysis Protocols (PRISMA‐P)^4^ and registered in PROSPERO on April 28, 2020 (CRD42020173348).

We searched for RCTs, including cluster‐randomized studies, on pregnant women and their offspring. The primary target settings were South Asia and Sub‐Saharan Africa, but other settings were not excluded. Interventions included supplementary calcium alone or with vitamin D during pregnancy, starting at 35 gestational weeks at the latest and stopping at delivery. We accepted studies using elemental calcium doses up to the maximum of 2000 mg/day and vitamin D (vitamin D, cholecalciferol, or ergocholecalciferol) doses up to the maximum of 4000 IU (100 μg)/day. Studies on food fortification were excluded. We included studies with control groups using placebo, no treatment, standard care, or regular diet.

The originally chosen follow‐up periods for maternal bone health were during pregnancy and up to 2 years after delivery, and in the case of offspring, from birth up to the age of 7 years. Since the number of studies was scarce, eventually even longer follow‐up periods were accepted. Primary outcomes of interest for maternal bone health were BMD, bone area (BA), and bone mineral content (BMC) measured in the whole body, lumbar spine, hip, radius, or tibia. Primary outcomes of interest for offspring's bone health were BMD, BMC, BA, bone mass, and bone mineral accretion measured in the whole body, lumbar spine, hip, radius, or tibia, and the incidence of rickets. Both maternal and offspring secondary outcomes were teeth firmness, with the variables including dental caries, decayed teeth, missing teeth, filled teeth, erupted permanent second molars, enamel hypoplasia, and teeth mineral density. Mixed dentition was studied only in the offspring and bone fractures only in the mother.

### Search strategy

An experienced information specialist (J.I.) developed the literature search strategy. Searches were conducted in MEDLINE (via OvidSP), Cochrane Central Register of Controlled Trials (CENTRAL, via Wiley Cochrane Library), CINAHL Complete (via EbscoHOST), Scopus, Science Citation Index (via Web of Science), Social Science Citation Index (via Web of Science), Conference Proceedings Citation Index ‐ Science (via Web of Science), Conference Proceedings Citation Index ‐ Social Science & Humanities (via Web of Science), ClinicalTrials.gov, WHO International Clinical Trials Registry Platform (WHO ICTRP), and PROSPERO.

Strategies for identifying relevant reports of RCTs comprised both database‐specific subject headings (e.g., Medical Subject Headings (MeSH)) and terms that are likely to appear in study titles and abstracts (Supplementary File 1, online only). We applied published search filters to inform the strategies in MEDLINE^5^ and CINAHL.^6^

We did not include animal studies. Publication types unlikely to provide relevant information, such as editorials and news, were excluded where possible. No other limits were applied. We screened the reference lists of relevant reviews for any additional applicable studies. No additional studies were identified.

Searches were undertaken on September 7, 2020. We loaded the results into EndNote (version X9.3, Clarivate Analytics) for deduplication.

### Study selection

Two authors (K.T. and P.K.) independently screened the titles and abstracts of the records, as well as full texts of studies eligible for outcome analysis using Covidence (Covidence systematic review software, Veritas Health Innovation, Melbourne, Australia), online software for streamlining systematic review processes. Disagreements were solved through discussion. Reasons for exclusion of the studies during full‐text screening were recorded. Neither of the review authors was blinded to the journal titles, study authors, or institutions. A PRISMA flow chart for the screening process was generated.

Next, two authors (K.T. and P.K.) independently extracted data using Covidence, solving disagreements by discussion. The extracted data included (1) author, publication year, country, and area; (2) study design; (3) characteristics of the study population and controls (number of randomized and analyzed, age, parity, and baseline dietary intake for calcium); (4) description and duration of intervention; (5) outcome measurements; and (6) effect measures, if applicable.

### Risk of bias

Two authors (K.T. and P.K.) independently evaluated the risk of bias by using the Cochrane risk‐of‐bias tool for randomized trials in Covidence with the following domains: sequence generation, allocation concealment, blinding of participants and personnel, blinding of outcome assessors, incomplete outcome data (attrition bias), selective outcome reporting, and other bias. Each domain was graded as having low, unclear, or high risk of bias, with disagreements solved by discussion. Studies with some concern as well as lack of information on the risk of bias were graded as having unclear risk of bias.^7^ Follow‐up reports on the same original study population were merged for the evaluation of risk of bias. For follow‐up studies or subsets of trials originally designed to evaluate the impact of calcium supplementation on hypertensive disorders in pregnancy, two risk‐of‐bias domains (sequence generation and allocation concealment) were assessed from the original trials. When appropriate, domains were assessed for different outcomes separately.

### Data analysis

The data that were extracted using Covidence were exported to RevMan 5.4 software (the Cochrane Collaboration, 2020) for further analyses. Continuous outcomes were analyzed using mean differences with 95% CI. We performed forest plot analyses for outcome parameters with the same measured bone site and approximately same time point from at least two different studies. We used fixed‐effect meta‐analysis for combining information where it was reasonable to assume that studies were estimating the same underlying treatment effect. The heterogeneity of the studies in the meta‐analysis was assessed using I^2^, a Chi‐squared test, and Tau^2^. In case of heterogeneity across the studies (I^2^ > 50%, Tau^2^ > 0 or *P* < 0.10), we used random‐effects meta‐analysis.

We planned to do subgroup analyses according to the dose of calcium (low dose: less than 1000 mg/day; high dose: 1000 mg or more/day), calcium supplementation alone and with vitamin D, women or populations with low dietary calcium intake (as defined by trial authors, or if not defined, mean daily intake less than 900 mg), geographical areas (Sub‐Saharan Africa and South Asia), teenagers and multiparas, if appropriate data were available.

### Interpretation of evidence

We interpreted the available evidence according to the coding as presented in Table 1.^8^ The categories included positive effect, possible positive effect, no positive effect, and unknown effect because of insufficient or inconclusive published research.

**Table 1:** Coding for the interpretation of the available evidence

Standardized statement	Situations included
Unknown effect: insufficient published research on the intervention's effect on the outcome	No RCTs, one low‐quality RCT with any result, or One moderate‐to‐high quality RCT where the 95% CI of the RR includes 1, or Only narrative reporting
Unknown effect: inconclusive published research on the intervention's effect on the outcome	At least two RCTs, 95% CI of the point estimate for an RR broadly spans both sides of 1 (ranges from <0.5 to >2)
Positive effect: the intervention likely reduces the risk of the adverse outcome	At least two moderate‐to‐high quality RCTs included in a meta‐analysis or IPD meta‐analysis, 95% CI of the point estimate of the RR is entirely less than 1
Possible positive effect: the intervention may reduce the risk of the adverse outcome	At least two RCTs included in a meta‐analysis or IPD meta‐analysis, 95% CI of the point estimate of the RR is entirely less than 1, but there is concern about the quality of the data, or At least two moderate‐to‐high quality RCTs included in a meta‐analysis or IPD meta‐analysis, 95% CI of the point estimate of the RR includes 1 but the 90% CI of the point estimate of the RR is entirely less than 1, or One moderate‐to‐high quality RCT, 95% CI of the point estimate of the RR is entirely less than 1
No positive effect: the intervention is unlikely to reduce the risk of the adverse outcome	Other situations, including meta‐analysis results suggestive of harm

CI, confidence interval; IPD, individual participant data; RCT, randomized controlled trial; RR, relative risk.

## Results

### Study selection

**Figure 1:**
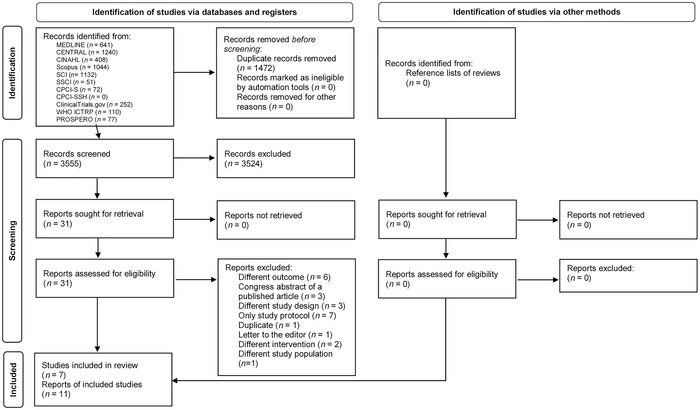
Selection of the randomized controlled trials to the review. Figure is modified from Ref. 26.

Literature searches retrieved 5027 records in total, and 3555 records remained for assessment of titles and abstracts after deduplication. Out of these, 3524 records were excluded, and full text screening of the remaining 31 references for eligibility was performed. Altogether seven RCTs (11 reports) involving 1566 participants (*n* = 413 women with maternal outcome data and *n* = 1153 with offspring outcome data) were included in this review (Fig. 1).

### Study characteristics

The included studies were published between 1978 and 2019 and seven of the reports were published in 2010 or later. From the seven identified studies (Table 2), three were trials originally examining the prevention of hypertensive disorders during pregnancy by calcium supplementation and including more than 6000 participants.^9^, ^10^, ^11^ Four studies, including 531 participating mothers, were originally designed to establish the effect of calcium supplementation during pregnancy on bone density.^12^, ^13^, ^14^, ^15^

**Table 2:** Characteristics of the included randomized controlled studies

Original reference	Raman *et al*.^12^	Belizán *et al*.^9^	Levine *et al*.^10^	Wang *et al*.^13^	Diogenes *et al*.^14^	Goldberg *et al*.^11^		Cullers *et al*.^15^
Country	India	Argentina	The United States	China	Brazil	The Gambia		The United States
Number of originally randomized women (Ca/placebo), population	273, study groups not specified, poor socioeconomic segment of the population	593/601, selected hospitals	2295/2294, nulliparous	55/55, selected hospital area	43/41, adolescents (13–19 years old) in select hospital area	330/332, rural villages		32/32
Baseline maternal calcium intake mg/day (mean ± SD), Ca/placebo	NA	646 ± 396/ 642 ± 448	1113 ± 691/ 1135 ± 675	NA (600–800 in average in China)	500 ± 276/ 743 ± 457	355 ± 190 (whole study population)		708 ± 380/ 757 ± 323
Daily dose of calcium and vitamin D	Group 1:300 mg Ca Group 2:600 mg Ca Group 3: placebo	2000 mg Ca	2000 mg Ca	300 mg Ca + 400 IU vitamin D	600 mg Ca + 200 IU vitamin D	1500 mg Ca		1000 mg Ca
Intervention started	18–22 weeks of gestation	20 weeks of gestation	13–21 weeks of gestation	Mid‐pregnancy	26 weeks of gestation	20 weeks of gestation		16 ± 2 weeks of gestation
Original primary outcome	Maternal and neonatal bone density in peripheral bones	Incidence of hypertensive disorders of pregnancy	Incidence of preeclampsia	Maternal radial BMD and neonatal BMD of tibia and fibula	Maternal and neonatal bone mass, fetal growth	Maternal systolic and diastolic blood pressure at 36 weeks of gestation		Peripheral cortical and trabecular bone changes during a reproductive cycle
Maternal outcome reference	Raman *et al*.^12^			Wang *et al*.^13^	Diogenes *et al*.^14^	Jarjou *et al*.,^20^ Jarjou *et al*.^21^		Cullers *et al*.^15^
Time points and measured bone sites (number of women data obtained Ca/placebo)	Not stated specifically, second metacarpal, fourth metacarpal, and first phalanx. (group 1:24, group 2:25, and group 3:38)			From mid‐ to late‐term pregnancy to delivery, radius (31/79)	5 weeks pp (26/21), 20 weeks pp (26/21), total body, lumbar spine, total hip, and femoral neck	2 weeks pp (24/27), 52 weeks pp (34/31), NPNL (30/28), F52 (22/20), whole body, lumbar spine, 2 weeks pp (20/23), 52 weeks pp (34/31), NPNL (30/28), F52 (22/20), total hip, femoral shaft, trochanter, femoral neck, 2 weeks pp (56/60), distal radius, and midshaft radius		16 gw (32/32), 36 gw (26/27), 4 months pp (22/23), 12 months pp (15/15), tibial distal metaphyseal and proximal diaphyseal site, radial distal metaphyseal and proximal diaphyseal site
Offspring outcome reference	Raman *et al*.^12^	Bergel *et al*.^16^	Koo *et al*.^17^	Wang *et al*.^13^	Diogenes *et al*.^18^	Jarjou *et al*.^19^	Ward *et al*. (2017)^22^	
Time points and measured bone sites (number of children data obtained Ca/placebo)	Neonatal, radius, ulna, tibia, and fibula, (group 1:24, group 2:25, and group 3:38)	12 years old, primary and permanent teeth (98/97)	Within first week, total body lumbar spine (128/128)	Neonatal, tibia, fibula (after randomization, significant shift from intervention to the control group, 31/79)	5 weeks pp, total body (30/26)	2 weeks pp (20/24), 13 weeks pp (27/20), 52 weeks pp (24/28), whole body 2 weeks pp (60/62), 13 weeks pp (57/55), 52 weeks pp (51/48), radius	8–12 years old, whole body, lumbar spine, and total hip (225/224)	
Technique	X‐ray	Clinical examination	DXA	SPA	DXA	DXA (whole body, lumbar spine, total hip), SPA (midshaft radius), and pQCT (tibia)	DXA	pQCT

NPNL indicates in the study by Jarjou *et al*.^21^ that the same women as at 52 weeks pp were invited for follow‐up when neither pregnant nor lactating for >3 months. F52 indicates in the study by Jarjou *et al*.^21^ that the same women as at 52 weeks pp were invited for follow‐up in a future lactation.

NA, not available; gw, gestational weeks; pp, postpartum; DXA, dual‐energy X‐ray absorptiometry; SPA, single‐photon absorptiometry; pQCT, peripheral quantitative computed tomography.

Two studies were conducted in the United States, while the remaining were from middle‐ or low‐income countries, that is, India, China, the Gambia, Brazil, and Argentina. One study was conducted specifically in pregnant women in the poor socioeconomic segment^12^ and one in adolescent women;^14^ otherwise, participants were unselected pregnant women in defined areas or populations. The numbers of participants ranged between 64 and 447 per reports (Table 2). The mean maternal daily nutritional calcium intake was under the recommended daily allowance (<900 mg) in three studies,^11^, ^14^, ^15^ within recommendation in one,^10^ and in three studies, it was not clearly stated.^12^, ^13^, ^16^

Three studies used low‐dose calcium (<1000 mg) and four studies used high‐dose calcium (≥1000 mg). Two studies combined vitamin D (200–400 IU = 5–10 μg) with low‐dose calcium supplementation. Intervention started before the third trimester of pregnancy and ended at parturition in all studies (Table 2). Maternal compliance was stated in 6 out of 11 reports. The compliances ranged between from 62%^17^ to 85%^14^, ^18^ to nearly 100%.^19^, ^20^, ^21^

Six studies reported maternal or offspring bone outcomes, and one evaluated offspring oral health. No studies reported rickets, bone fractures, or maternal oral health as outcomes. Maternal bone outcomes were measured from peripheral bones (metacarpal, phalanx, radius, or tibia) in four studies, and two studies included whole body, lumbar spine, total hip, or femoral neck. Three studies included follow‐up during lactation. There were no significant differences in the percentages of women exclusively or predominantly breastfeeding between the study groups when data were available.^14^, ^20^, ^21^ For offspring, peripheral bones (radius, ulna, tibia, or fibula) were measured in three studies, and whole body, lumbar spine, or total hip in four studies. Three studies included a follow‐up period after the neonatal period, up to 12 years of age. The child's calcium or vitamin D intake was not specified in these reports.^18,19,22^ One study in Argentina evaluated offspring oral health at 12 years of age.^16^ The first measurement point for bone outcomes after pregnancy and the follow‐up period varied across the studies, as well as techniques to estimate bone mineralization (Table 2).

### Risk of bias

**Figure 2:**
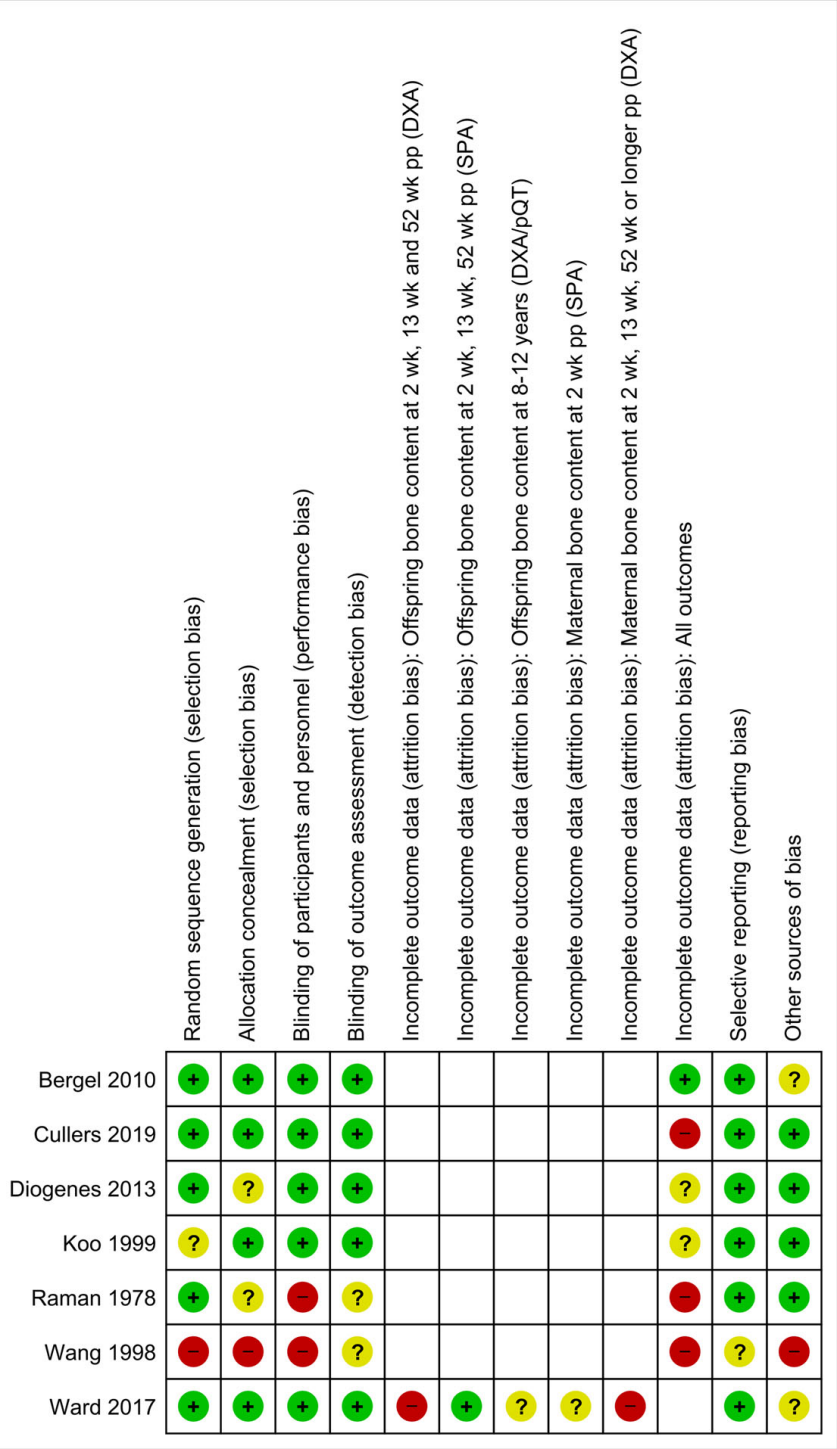
Risk of bias summary: review authors' judgments about each risk of bias item for each included study.

Five out of seven studies were assessed as having a low risk of bias in at least five of seven evaluated domains. And two studies were graded as having unclear or high risk of bias in over half of the evaluated domains (Fig. 2).

The risk of bias was estimated to be low considering random sequence generation, allocation concealment, blinding of participants, personnel, and outcome assessment domains in five out of seven studies (Fig. 2). In three studies, there were no clear statements of allocation concealment^12^, ^14^ or random sequence generation,^17^ and in one study, the intervention was not blinded,^12^ leading to a high risk of bias. A study conducted in China was considered to have high risk of attrition bias, since after randomization, 44% of women randomized to intervention group were able to shift to the placebo group.^13^

One of the major concerns was incomplete outcome data. The amount of missing data depended on the technique used and follow‐up periods, but it was high in nearly all reports: 68%,^12^ 17–30%,^15^ 48–66%,^21^ 32%,^22^ 58–65%,^19^ 44%,^14^ 33%,^18^ and 44%.^13^ Furthermore, statements of sample size calculations were found in only a few reports.^14^, ^15^, ^19^

The risk of selective reporting bias was estimated to be low in six studies and unclear for one study^13^ since the statements on primary and secondary outcomes were missing. In the studies having the longest follow‐up periods, there was a lack of data on potentially confounding or effect‐modifying factors, such as nutritional intake, economic and educational factors, or physical activity, which may have an impact on outcomes of bone and oral health; therefore, the risk of bias was estimated to be unclear.^16^, ^21^, ^22^

### Maternal bone outcomes

#### Bone health during pregnancy

BMD during pregnancy was evaluated in three studies (*n* = 447 randomized women; Table 3).^12^, ^13^, ^15^ Two studies from India and China used low‐dose (either 300 or 600 mg) calcium supplementation in women with low calcium intake; the latter combined calcium supplementation with vitamin D. One study conducted in the United States used high‐dose calcium supplementation (1000 mg) in women with low calcium intake. All studies included high risk of bias—especially due to missing data.

**Table 3:** The effect of calcium supplementation during pregnancy on maternal bone health, in comparison with placebo

Study	Raman *et al*.^12^	Wang *et al*.^13^	Diogenes *et al*.^14^		Jarjou *et al*.,^20^ Jarjou *et al*.^21^				Cullers *et al*.^15^		
Time point	Mid‐ to late‐term pregnancy	Mid‐ to late‐term pregnancy to delivery	5 weeks pp	20 weeks pp	2 weeks pp	52 weeks pp	NPNL	F52	36 gw	4 months pp	12 months pp
Bone site	Second and fourth metacarpal, first phalanx BMD ↑*a*	Radius BMD ↑	Whole body BMD NS BMC NS BA NS	Whole body BMD NS BMC NS BA NS	Whole body BMD NS BMC NS BA NS	Whole body BMD ↓ BMC ↓ BA ↓	Whole body BMD ↓ BMC ↓ BA ↓	Whole boby BMD ↓ BMC ↓ BA ↓	Radial distal*b* tot vBMD NS trab vBMD NS	Radial distal*b* tot vBMD NS trab vBMD NS	Radial distal*b* tot vBMD NS trab vBMD NS
Bone site			Lumbar spine BMD NS BMC NS BA ↑	Lumbar spine BMD NS BMC NS BA NS	Lumbar spine BMD NS BMC NS BA NS	Lumbar spine BMD ↓ BMC ↓ BA NS	Lumbar spine BMD ↓ BMC ↓ BA NS	Lumbar spine BMD ↓ BMC ↓ BA NS	Radial proximal*c* tot vBMD NS cor vBMD NS	Radial proximal*c* tot vBMD NS cor vBMD NS	Radial proximal*c* tot vBMD NS*d* cor vBMD NS
Bone site			Total hip BMD NS BMC NS BA NS	Total hip BMD NS BMC NS BA NS	Total hip BMD ↓ BMC ↓ BA ↓	Total hip BMD ↓ BMC ↓ BA ↓	Total hip BMD ↓ BMC ↓ BA ↓	Total hip BMD ↓ BMC ↓ BA ↓	Tibial distal*b* tot vBMD NS trab vBMD NS	Tibial distal*b* tot vBMD NS trab vBMD NS	Tibial distal*b* tot vBMD NS trab vBMD NS
Bone site			Femoral neck BMD NS BMC NS BA NS	Femoral neck BMD NS BMC NS BA NS	Femoral neck BMD NS BMC NS BA NS	Femoral neck BMD ↓ BMC ↓ BA NS	Femoral neck BMD ↓ BMC ↓ BA ↓	Femoral neck BMD ↓ BMC ↓ BA NS	Tibial proximal*c* tot vBMD NS cor vBMD NS	Tibial proximal*c* tot vBMD NS cor vBMD NS	Tibial proximal*c* tot vBMD NS cor vBMD NS*d*
Bone site					Femoral shaft BMD ↓ BMC ↓ BA NS	Femoral shaft BMD ↓ BMC ↓ BA ↓	Femoral shaft BMD ↓ BMC ↓ BA ↓	Femoral shaft BMD ↓ BMC ↓ BA ↓			
Bone site					Femoral trochanter BMD NS BMC NS BA NS	Femoral trochanter BMD ↓ BMC ↓ BA ↓	Femoral trochanter BMD ↓ BMC ↓ BA NS	Femoral trochanter BMD ↓ BMC ↓ BA ↓			
Bone site					Distal radius BMD NS BMC NS BA NS						
Bone site					Midshaft radius BMD NS BMC NS BA NS						

NPNL indicates in the study by Jarjou *et al*.^21^ that the same women as at 52 weeks pp were invited for follow‐up when neither pregnant nor lactating for >3 months. F52 indicates in the study by Jarjou *et al*.^21^ that the same women as at 52 weeks pp were invited for follow‐up in a future lactation.

NS, no significant differences between the study groups; ↑, significantly higher in the intervention group compared with the placebo, *P* ≤ 0.05; ↓, significantly lower in the intervention group compared with the placebo, *P* ≤ 0.05.

^*a*^ In the highest supplementation group (600 mg): ↑ in the fourth metacarpal.

^*b*^ Bone density was assessed from a distal metaphyseal site of tibia or radius.

^*c*^ Bone density was assessed from a proximal diaphysis site of tibia or radius.

^*d*^ No difference at 12 months after delivery, but statistically significant group × time interaction effects were noticed from baseline to 12 months pp (visit 5) for two variables (radial diaphyseal total BMD: *P* = 0.029; tibial diaphyseal cortical BMD: *P* = 0.015).

BMC, bone mineral content (g); BA, bone area (cm^2^); cor, cortical; pp, postpartum; tot, total; trab, trabecular; (v)BMD, (volumetric) bone mineral density (g/cm^2^ or g/cm^3^).

In the high‐dose supplementation study, there were no differences between the study groups in volumetric BMDs (vBMDs) in radial and tibial distal and proximal sites.^15^ In the low‐dose calcium supplementation studies, insignificant differences were found in most measured bone sites, except radial^13^ and one metacarpal (in the highest supplement dose, i.e., 600 mg)^12^ BMDs were found to be significantly higher in the intervention group during pregnancy or around delivery.

#### Bone health within weeks after delivery

Two studies on a total of 209 randomized women evaluated BMDs within weeks after delivery (Table 3). A study from Brazil used low‐dose calcium (600 mg) with vitamin D^14^ and another from the Gambia used high‐dose calcium supplementation (1500 mg) in women with low calcium intake.^20^ The estimated risk of bias due to missing data ranged from high to unclear in these studies.

No significant differences were found in BMDs of the whole body, lumbar spine, or femoral neck.^14^, ^20^ Meanwhile, BMDs in total hip and femoral shaft were found to be significantly lower in the intervention group in the Gambian study. No significant differences were found in radial BMDs.^20^

#### Bone health during breastfeeding or later

Three studies on a total of 273 randomized women estimated the impact of maternal calcium supplementation on her BMD during breastfeeding (Table 3). Baseline maternal calcium intake was lower than recommended in all the study populations. The dose of calcium supplementation was low with vitamin D in the Brazilian study^14^ and high in studies conducted in the United States^15^ and the Gambia.^21^ All of these studies included unclear or high risk of bias due to incomplete outcome data.

Two studies found no statistically significant differences in BMDs of the whole body, lumbar spine, total hip, and femoral neck,^14^ or vBMDs of radius and tibia at 4 months to 1 year after delivery.^15^ However, in both of these studies, some positive effect of intervention was shown as a slower rate of losing BMD during follow‐up in the intervention group. In the Brazilian study, significantly less femoral neck BMD loss was observed in the calcium supplementation group between 5 and 20 weeks after delivery.^14^ Similarly, the decrease of bone content from 36 gestational weeks to 12 months after delivery in total vBMD at radial proximal diaphyseal as well as in cortical vBMD at the tibial proximal diaphyseal site was less in the intervention group.^15^

By contrast, in the Gambian study, the BMDs in the whole body, lumbar spine, total hip, and femoral sites were significantly lower in the calcium supplementation group at 1 year after delivery.^20^ The significant difference persisted in two long follow‐up periods: nonpregnant mothers ≥3 months after the stop of lactation, and in mothers at 52 weeks after delivery in a subsequent lactation.^21^

**Figure 3:**
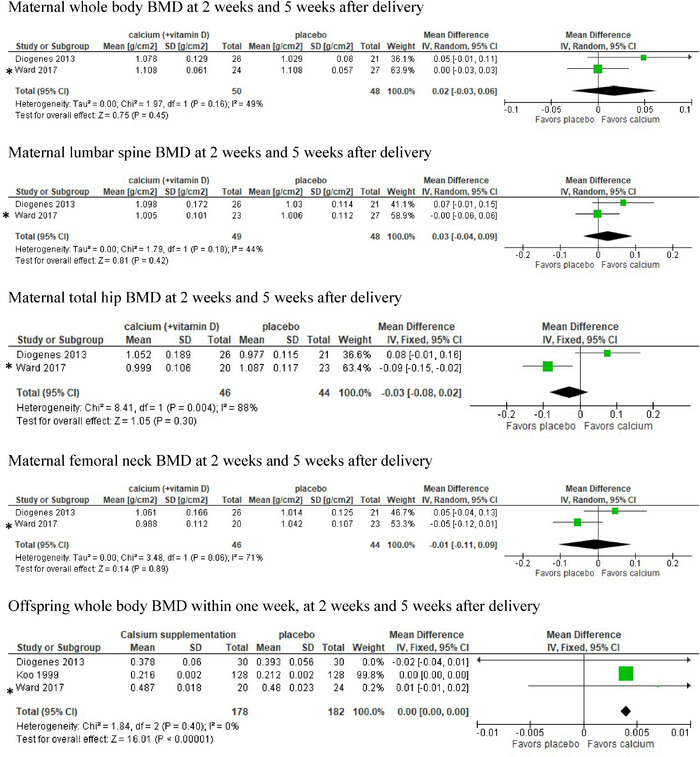
Meta‐analysis and forest plots of the studies of maternal and offspring bone mineral density after delivery. The reference citations for the indicated studies are Diogenes 2013 (Ref. 14), Ward 2017 (Ref. 22), and Koo 1999 (Ref. 17). ^*^ Owing to the merging of studies, Ward 2017 refers to the report by Jarjou 2006 (Ref. 19) when considering offspring outcome and the report by Jarjou 2013 (Ref. 21) considering maternal outcomes. [Correction added on December 06, 2021, after first online publication: In Figure 3, placement of the labels “favors placebo” and “favors calcium” was corrected.]

A meta‐analysis that included two studies on maternal BMDs in the whole body, lumbar spine, total hip, and femoral neck at 2–5 weeks after delivery did not favor either group.^14^, ^20^ The heterogeneity was substantial (I^2^ > 30%) (Fig. 3).

The impact of maternal calcium supplementation during pregnancy on the mother's bone health during pregnancy, after delivery, and during and after lactation was deemed unknown because of inconclusive research results.

### Offspring bone outcomes

#### Bone health during the neonatal period

There were three studies including a total of 453 infants on bone outcomes during the neonatal period (Table 4).^12^, ^13^, ^17^ One study was from India^12^ on 87 mothers with either 300 or 600 mg/day calcium doses or placebo, one study was from China^13^ on 110 mothers with 300 mg/day calcium combined with vitamin D in the intervention group, and one was a substudy from the trial on preeclampsia prevention in the United States.^17^ The American study included 256 mothers with high dose (2000 mg/day) calcium supplementation as an intervention in women with sufficient calcium intake. All these studies were assessed as having unclear or high risk of bias.

**Table 4:** The effect of calcium supplementation during pregnancy on offspring bone health, in comparison with placebo

Study	Raman *et al*.^12^	Wang *et al*.^13^	Koo *et al*.^17^	Diogenes *et al*.^18^	Jarjou *et al*.^19^			Ward *et al*.^22^
Time point	Neonatal	Neonatal	Within the first week	5 weeks	2 weeks	13 weeks	52 weeks	8–12 years
Whole body			BMD NS BMC NS BA NS	BMD NS BMC NS BA NS	BMD NS BMC NS BA NS	BMD NS BMC NS BA NS	BMD NS BMC NS*b* BA NS*b*	BMC ↓ F/NS M ns F/NS M BA ↓ F/NS M*c* ns F/NS M*d*
Lumbar spine			BMD NS BMC NS BA NS					BMC ↓ F/NS M NS F/NS M BA NS F/NS M*c* NS F/NS M*d*
Total hip								BMC ↓ F/NS M NS F/NS M BA ↓ F/NS M*c* NS F/NS M*d*
Midshaft radius/radius/ulna/tibia/fibula	BMD ↑*a*	BMD ↑			BMD NS BMC NS BA NS	BMD NS BMC NS BA NS	BMD NS BMC NS BA NS	tot vBMD NS F/NS M*c* NS F/NS M*d* BMC NS F/NS M*c* NS F/NS M*d*

NS, no significant differences between the study groups; ↑, significantly higher compared with the placebo, *P* ≤ 0.05; ↓, significantly lower compared with the placebo, *P* ≤ 0.05.

^*a*^ Considering both studied calcium supplementation groups (300 and 600 mg).

^*b*^ Significantly slower increase in BMC and BA between the age of 2 and 52 weeks in the calcium supplementation group.

^*c*^ In the study by Ward *et al*.,^22^ two models were constructed to test for sex*supplement effects on the growth of the children at 8–12 years. The first model included length at 52 weeks, current age, sex (M/F), maternal supplement group (Ca/P), and a sex*supplement group interaction.

^*d*^ In the study by Ward *et al*.,^22^ two models were constructed to test for sex*supplement effects on the growth of the children at 8–12 years. The second model was based on the first but adjusted the bone and body composition data for current body size, using height and weight for bone variables and height for lean and fat masses.

BA, bone area (cm^2^); BMC, bone mineral content (g); BMD, bone mass density (g/cm^2^); F, female; M, male; tot vBMD, total volumetric BMD (g/cm^3^).

No intergroup differences were found in the American study, either in whole‐body or lumbar spine BMD. In a further analysis, the study population was divided according to maternal dietary calcium intake at baseline. Among neonates of mothers with calcium intake in the lowest quintile (mean = 411 mg/day, *n* = 51 infants), the calcium supplementation group had significantly higher mean whole‐body BMC (64.1 g) compared with the placebo (55.7 g) group during the first week of life. No significant differences were found in other calcium intake quintiles.^17^ In two smaller studies with a high risk of bias, the BMDs of the infants were significantly higher in the supplementation groups in peripheral bones, that is, radius, ulna, tibia, and fibula.^12^, ^13^

#### Bone health within weeks after birth

Later, within weeks after birth, BMDs were established in two studies (Table 4), one from Brazil (56 offspring of 84 adolescent mothers using low‐dose calcium with vitamin D versus placebo)^17^ and one from the Gambia (42–120 infants of 662 mothers using high‐dose calcium versus placebo).^19^ Maternal baseline calcium intake was low in both studies. Both studies included some risk of bias because of missing data.

No significant differences between the study groups were found in BMDs measured from the whole body^17^, ^19^ or radius^19^ within 2–13 weeks after delivery. The finding was similar in whole‐body and radial BMDs at the subsequent follow‐up at 52 weeks after birth in the Gambian study.^19^ A further analysis suggested a slower rate of increase of whole‐body BMC and BA, but not whole‐body BMD, within the calcium supplement group, suggesting a slower velocity of bone growth at 52 weeks compared with the placebo group.^19^

#### Bone health in later childhood

The longest follow‐up study of 8–12 years was on 499 children from the Gambia,^22^ where the original maternal calcium dose had been 1500 mg/day (Table 4). There was unclear risk of bias in the category of other sources bias, since confounding factors, such as nutrition (especially dietary calcium intake), education, and income, which may influence the bone content outcomes in long‐term follow‐ups, were not stated.

From this analysis, comparisons were only reported separately for females and males. In females, the BMCs and BAs in the whole body, lumbar spine (BMC only), and total hip were lower in the intervention group compared with the controls, when adjusted for length at 1 year of age. Among females, the studied bones were also lighter and smaller (lower BMC and BA in the whole body, lumbar spine, and total hip) in the intervention group than the control group at 8–12 years of age. However, the difference became nonsignificant when the analysis was adjusted for the participants’ current body size (height and weight). No differences were found in peripheral bones (tibia) in any analyses.^22^

A meta‐analysis of offspring whole‐body BMD measured within weeks after birth included three studies.^17^, ^18^, ^19^ It very marginally favored calcium suppplementation (Fig. 3). [Correction added on December 06, 2021, after first online publication: The last sentence of this paragraph was corrected.]

The impact of maternal calcium supplementation during pregnancy on offspring bone health was deemed unknown because of inconclusive research results.

#### Offspring dental health

One trial from Argentina,^16^ where the high‐dose calcium supplementation (2000 mg/day) or placebo was originally aimed to prevent hypertensive disorders in pregnancy (*n* = 1194 women), conducted a follow‐up study on offspring oral health. A total of 195 children at the age of 12 years were examined. The risk of bias was unclear in the category of other sources of bias, since differences in the study population characteristics, such as nutrition (especially calcium intake), social situation, or income, which potentially lead to bias, were not taken into account.

The numbers of children with at least one decayed, missing, or filled tooth in the permanent and primary teeth (DMFT/dmft) were significantly lower in the intervention group (*n* = 62) than the control group (*n* = 84) (63% versus 87%, *P* < 0.001). When these were estimated separately for the primary and permanent teeth, the significant difference remained only with permanent teeth (*n* = 59 (60%) versus *n* = 79 (81%), *P* < 0.001). The numbers of decayed, missing, or filled surfaces (DMFS/dmfs) were significantly lower in the calcium supplementation group compared with the placebo group (mean (SD) = 3.1 (4.1) versus 4.4 (4.1), *P* < 0.001). No differences were found in the numbers of children with erupted permanent second molars, with mixed dentition, or with enamel hypoplasia. Our interpretation of the data was that maternal calcium supplementation during pregnancy possibly improves dental health of the offspring during childhood.

## Discussion

### Summary of main results

#### The effect of calcium supplementation during pregnancy on maternal bone health

In our systematic review, we found inconclusive results for calcium supplementation on maternal BMD during mid‐ to late pregnancy and after the stop of the intervention within weeks after delivery.^12^, ^13^, ^14^, ^15^, ^20^ This was because of heterogeneity of the assessment methods, risks of bias, and partly the low quality of the studies. Also, meta‐analyses on maternal BMDs up to 5 weeks postpartum did not favor either group. The effect of calcium supplementation was not enhanced either by a high dose of calcium or concomitant vitamin D.

Later, about 1 year after delivery and during lactation, one study^20^, ^21^ found that the results of the maternal skeletal assessments were worse in the calcium supplementation group compared with placebo. No improvement was found after the cessation of lactation and in a subsequent lactation.^21^

#### The effect of maternal calcium supplementation during pregnancy on offspring bone health

Some better BMDs in the maternal calcium supplementation group compared with the controls during the early neonatal period were suggested in some studies.^12^, ^13^ A meta‐analysis of offspring BMD within weeks after birth very marginally favored calcium supplementation, but the summary of the follow‐up study up to 1 year of age showed no significant advantage of the intervention on offspring bone health.^19^ The effect of calcium supplementation during pregnancy on the offspring BMDs was deemed to be unknown because of inconclusive research results. This was mainly because of high or unknown risk of bias due to incomplete outcome data. [Correction added on December 06, 2021, after first online publication: The second and third sentences of the previous version of this paragraph were corrected and combined into the second sentence of the current version of this paragraph.]

One study reported a slower bone growth velocity during the first year of life in the calcium supplementation group.^19^ In addition, a long‐term follow‐up study showed a sex‐specific difference, that is, a slower growth and smaller skeleton size and composition at 8–12 years of age in female offspring of calcium‐supplemented mothers.^22^

Maternal high‐dose calcium supplementation may possibly have a long‐term positive effect on the teeth firmness of offspring.^16^

### Completeness and applicability of evidence

The amount of missing data was notable, being 68% at the highest,^12^ and between 17% and 30% at the lowest.^15^ The primary objective of three of the included studies, involving the majority of study subjects in this review, was hypertensive disorders in pregnancy,^9^, ^10^, ^11^ while the smaller proportion of the subjects were from studies where maternal and/or offspring bone health was the primary outcome measure.^12^, ^13^, ^14^, ^15^ Because the original study populations included participants from low‐, middle‐, and high‐income countries, and with both low and sufficient maternal dietary calcium intakes, the applicability of the results cannot be specified for any particular populations, dietary patterns, or geographic areas.

### Quality of evidence

The numbers of participants were small, that is, altogether less than a hundred participants in each group in the studies on maternal bone health.^12^, ^13^, ^14^, ^15^, ^20^, ^21^ In the studies concerning offspring bone health, the numbers of participants ranged between 350 and 450, depending on the ages at assessment.^12^, ^13^, ^17^, ^18^, ^21^, ^22^ Clear statements of allocation concealment or random sequence was missing in almost half of the studies,^12^, ^14^, ^17^ and the intervention was not blinded in one.^12^ The quality of one study was hampered by allowing a shift from the intervention group to the placebo after randomization and another due to an unclear selective reporting bias.^13^ Sample size calculations were appropriately specified in less than half of the reports.^14^, ^15^, ^19^

Lack of data on effect‐modifying factors, including dietary habits, nutrient and vitamin intakes, and physical activity, which may have an impact on bone and oral health, was a weakness, especially in the studies with longest follow‐up periods.^16^, ^21^, ^22^

### Potential biases in the review process

The searches were thoroughly planned in close collaboration with an experienced information specialist who also performed the searches. Criteria of inclusion of studies were clearly defined. The members of the review team were not involved in any of the studies. The review process was performed according to the PRISMA‐P. Post‐hoc inclusions were defined and reported.

### Agreements and disagreements with other studies or reviews

Although calcium supplementation during pregnancy has been shown to be beneficial in preventing preeclampsia,^23^, ^24^ there was no clear evidence of any sustained benefit of the supplementation on maternal bone health. Instead, possible long‐term adverse effects on maternal bone health after withdrawal of the supplementation are of concern. The investigators of the study from the Gambia proposed that pregnant women with low dietary calcium intake might have adaptive mechanisms for the maintenance of the bone mineralization. High‐dose calcium supplementation during pregnancy might downregulate this adaptation for a prolonged time. After withdrawal of the supplementation, this might lead to reduced bone density during lactation, after weaning from breastfeeding, and in a subsequent lactation.^20^, ^21^ More studies are needed to confirm this finding.

A recently published systematic review assessed whether calcium supplementation has a beneficial effect on maternal BMD during lactation.^25^ Their search yielded five RCTs, including 567 women. Calcium supplementation had started at 36 weeks of gestation in one study, and after delivery in the remaining four studies. The conclusion was that calcium supplementation seems not to be useful for improving maternal BMD during lactation. The studies were very heterogeneous in terms of dietary calcium intake, calcium dosage, and combination with vitamin D. Thus, the quality of evidence was low. The focus of their review did not match with the focus of our analysis, making any comparisons with our results impossible.

Earlier reviews on maternal calcium supplementation during pregnancy on offspring BMD or dental health were not available.

## Conclusions

### Implications for practice

We did not find any evidence of benefit of calcium supplementation during pregnancy without or with vitamin D on maternal or offspring bone density after delivery, or later during breastfeeding. Some long‐term adverse effects in maternal bone firmness and growth of female offspring are even suspected. The only positive finding of high‐dose calcium supplementation was a possible long‐term benefit on offspring teeth firmness.

Our ability to draw firm conclusions on the impact of calcium on the studied outcomes is reduced by the few numbers of studies, low numbers of participants, and high amount of missing data in included studies. The included studies were also heterogeneous in terms of baseline dietary calcium intake, duration of pregnancy at start of intervention, duration of supplementation, calcium dosage, and combination with vitamin D. The quality of evidence is low, because of insufficient research data. Thus, the results should be interpreted with caution.

### Implications for research

Data on the effects of calcium supplementation during pregnancy on maternal bone density during lactation are limited and the suspected harmful effects need to be either confirmed or refuted. If confirmed, the recovery of the adaptive mechanisms of calcium metabolism during pregnancy and after delivery needs to be studied further. Further research is also needed in order to establish whether calcium supplementation started during pregnancy and continued uninterrupted during lactation would be beneficial for maternal and offspring bone firmness.

Further research is also needed to provide evidence regarding long‐term benefits or harms of the intervention on outcomes, such as maternal osteoporosis. In addition, more studies are needed on bone and dental health of infants and children of calcium‐supplemented mothers.

## Author Contributions

K.T. participated in designing and writing the study protocol, screened the studies, and had primary responsibility in analyzing the data and writing the manuscript. P.K. participated in designing and writing the study protocol, screened the studies, and participated in analyzing the data and writing the manuscript. J.I. performed the literature searches and participated in writing the manuscript. R.O. participated in designing and writing the study protocol and revising the manuscript. U.A. participated in designing and writing the study protocol, interpreting the analyzed data, and writing the manuscript. P.A. participated in designing and writing the study protocol, interpreting the analyzed data, and writing the manuscript. O.T. participated in designing and writing the study protocol, interpreting the analyzed data, and writing the manuscript.

### Peer review

The peer review history for this article is available at https://publons.com/publon/10.1111/nyas.14705

## Competing interests

The authors declare no competing interests.

## Supporting information


**Supplementary File 1**. Search strategies used for the different literature databases.
