# Supplementary material for: Calcium supplementation during pregnancy and maternal and offspring bone health: a systematic review and meta‐analysis
Source: Ann N Y Acad Sci. 2021 Nov 15;1509(1):23–36. doi: 10.1111/nyas.14705 (PMC9298950; doi:10.1111/nyas.14705)
Supplement: Supplementary file 1 — Search strategies used for the different literature databases. [file NYAS-1509-23-s001.docx]

**Supplementary File 1.**

**1. Ovid MEDLINE(R) ALL**
Interface/URL: OvidSP
Database coverage dates: 1946 to September 04, 2020
Search date: 7 September 2020
Number of results retrieved: 641
Search strategy:
Note: The search filter developed by Cochrane (Cochrane Highly Sensitive Search Strategy for identifying randomized trials in MEDLINE: sensitivity- and precision-maximizing version (2008 revision) was applied (lines 10-17).
*Cochrane Highly Sensitive Search Strategy for identifying randomized trials in MEDLINE: sensitivity- and precision-maximizing version (2008 revision); Ovid format. In: Lefebvre C, Glanville J, Briscoe S, Littlewood A, Marshall C, Metzendorf M-I, Noel-Storr A, Rader T, Shokraneh F, Thomas J, Wieland LS. Technical Supplement to Chapter 4: Searching for and selecting studies. In: Higgins JPT, Thomas J, Chandler J, Cumpston MS, Li T, Page MJ, Welch VA (eds). Cochrane Handbook for Systematic Reviews of Interventions Version 6. Cochrane, 2019. Available from: www.training.cochrane.org/handbook*

1 exp Pregnancy/ (896223)

2 Pregnant Women/ (8396)

3 (pregnan$ or gestation$ or antenatal$ or ante-natal$ or prenatal$ or pre-natal$ or post-conception$ or postconception$).ti,ab,kf. (691093)

4 or/1-3 (1103723)

5 Calcium/ (267676)

6 Calcium, Dietary/ (14057)

7 calcium.ti,ab,kf. (381572)

8 or/5-7 (504668)

9 4 and 8 (12734)

10 randomized controlled trial.pt. (512469)

11 controlled clinical trial.pt. (93828)

12 randomized.ab. (491495)

13 placebo.ab. (210767)

14 Clinical Trials as Topic/ (192800)

15 randomly.ab. (340352)

16 trial.ti. (224546)

17 or/10-16 (1311438)

18 9 and 17 (871)

19 exp Animals/ not Humans/ (4731250)

20 18 not 19 (658)

21 (news or editorial or case reports).pt. or case report.ti. (2906723)

22 20 not 21 (656)

23 remove duplicates from 22 (641)

**2. Cochrane Central Register of Controlled Trials**Interface/URL: Wiley Cochrane Library
Database coverage dates: Issue 9 of 12, September 2020
Search date: 7 September 2020
Number of results retrieved: 1240
Search strategy:

#1 [mh Pregnancy] 21748

#2 [mh ^“Pregnant Women”] 258

#3 (pregnan* or gestation* or antenatal* or prenatal* or postconception* or ante NEXT natal* or pre NEXT natal* or post NEXT conception*) 75982

#4 #1 OR #2 OR #3 76187

#5 [mh ^”Calcium, Dietary”] 1546

#6 [mh ^calcium] 3399

#7 calcium 30312

#8 #5 OR #6 OR #7 30312

#9 #4 AND #8 in Trials 1240

**3. CINAHL Complete**Interface/URL: EBSCOhost
Database coverage dates: from inception to date
Search date: 7 September 2020
Number of results retrieved: 408
Search strategy:
Note: The search filter developed by Glanville et al was applied (lines S10-S27).
*Glanville J, Dooley G, Wisniewski S, Foxlee R, Noel-Storr A. Development of a search filter to identify reports of controlled clinical trials within CINAHL Plus. Health Info Libr J 2019 Mar;36(1):73-90. doi: 10.1111/hir.12251*

| **#** | **Query** | **Results** |
| --- | --- | --- |
| S30 | S26 NOT S29 | 408 |
| S29 | S27 NOT S28 | 187,015 |
| S28 | MH (Human) | 2,244,746 |
| S27 | (MH Animals+) OR (MH “Animal Studies”) OR TI (animal model*) | 214,923 |
| S26 | S9 AND S25 | 447 |
| S25 | S10 OR S11 OR S12 OR S13 OR S14 OR S15 OR S16 OR S17 OR S18 OR S19 OR S20 OR S21 OR S22 OR S23 OR S24 | 785,934 |
| S24 | AB (cluster W3 RCT) | 359 |
| S23 | MH (crossover design) OR MH (comparative studies) | 351,545 |
| S22 | AB (control W5 group) | 112,729 |
| S21 | PT (“randomized controlled trial”) | 122,175 |
| S20 | MH (placebos) | 12,865 |
| S19 | MH (“sample size”) AND AB (assigned OR allocated OR control) | 4,055 |
| S18 | TI (trial) | 109,468 |
| S17 | AB (random* | 315,995 |
| S16 | TI (randomised OR randomized) | 107,042 |
| S15 | (MH “cluster sample”) | 4,521 |
| S14 | (MH “pretest-posttest design”) | 43,687 |
| S13 | (MH “random assignment”) | 63,440 |
| S12 | (MH “single-blind studies”) | 14,250 |
| S11 | (MH double-blind studies) | 48,506 |
| S10 | (MH “randomized controlled trials”) | 108,227 |
| S9 | S4 AND S8 | 1,696 |
| S8 | S5 OR S6 OR S7 | 38,967 |
| S7 | TI calcium OR AB calcium | 31,019 |
| S6 | (MH "Calcium, Dietary") | 3,628 |
| S5 | (MH "Calcium") | 14,873 |
| S4 | S1 OR S2 OR S3 | 279,071 |
| S3 | TI (pregnan* or gestation* or antenatal* or prenatal* or postconception* or ante W3 natal* or pre W3 natal* or post W3 conception*) OR AB (pregnan* or gestation* or antenatal* or prenatal* or postconception* or ante W3 natal* or pre W3 natal* or post W3 conception*) | 178,905 |
| S2 | (MH “Expectant Mothers”) | 8,133 |
| S1 | (MH "Pregnancy+”) | 215,338 |

**4. SCOPUS**Interface/URL: http://www.scopus.com
Database coverage dates: from inception to date
Search date: 7 September 2020
Number of results retrieved: 1044
Search strategy:

( ( TITLE-ABS-KEY ( pregnan* OR gestation* OR antenatal* OR "ante-natal*" OR prenatal OR "pre-natal*" OR "post-conception*" OR postconception* ) AND TITLE-ABS-KEY ( calcium ) AND TITLE-ABS-KEY ( trial* OR random* OR "clinical stud*" OR "controlled stud*" ) ) ) AND NOT ( ( KEY ( animal* ) OR TITLE ( rat OR rats OR mice OR mouse OR hamster OR hamsters OR bovine OR sheep OR dog OR dogs OR cat OR cats OR rabbit OR rabbits OR calf OR calves OR cow OR cows OR pig OR pigs OR swine OR porcine ) ) AND NOT KEY ( human* ) ) AND NOT INDEX ( medline )

**5. Science Citation Index**Interface/URL: Web of Science
Database coverage dates: 1975-present
Search date: 7 September 2020
Number of results retrieved: 1132
Search strategy:

| # 7 | 1,132 | #5 NOT #6  *Indexes=SCI-EXPANDED Timespan=All years* |  |  |  |  |
| --- | --- | --- | --- | --- | --- | --- |
| # 6 | 2,382,400 | TI=("rat" or "rats" or "rodent" or "rodents" or "mouse" or "mice" or "murine" or "hamster" or "hamsters" or "gerbil" or "gerbils" or "animal" or "animals" or "dogs" or "dog" or "canine" or "pig" or "pigs" or "piglet" or "piglets" or "cat" or "cats" or "bovine" or "cow" or "cows" or "cattle" or "sheep" or "ewe" or "ewes" or "horse" or "horses" or "equine" or "ovine" or ”swine" or "porcine" or "monkey" or "monkeys" or "primate" or "primates" or "rhesus macaque" or "rhesus macaques" or "rabbit" or "rabbits") NOT TS=(human* or "women" or "woman" or "man" or "men" or "child" or "children" or adolescen* or teenager* or "people" or "boy" or "boys" or "girl" or "girls")  *Indexes=SCI-EXPANDED Timespan=All years* |  |  |  |  |
| # 5 | 1,283 | #3 AND #4  *Indexes=SCI-EXPANDED Timespan=All years* |  |  |  |  |
| # 4 | 2,611,496 | TS=(trial* OR random* OR "clinical" NEAR/3 stud* OR "controlled" NEAR/3 stud*)  *Indexes=SCI-EXPANDED Timespan=All years* |  |  |  |  |
| # 3 | 8,274 | #1 AND #2  *Indexes=SCI-EXPANDED Timespan=All years* |  |  |  |  |
| # 2 | 575,681 | TS=("calcium")  *Indexes=SCI-EXPANDED Timespan=All years* |  |  |  |  |
| # 1 | 596,766 | TS=(pregnan* OR gestation* OR antenatal* OR prenatal* OR postconception*) OR TS=(ante NEAR/3 natal* OR pre NEAR/3 natal* OR post NEAR/3 conception*)  *Indexes=SCI-EXPANDED Timespan=All years* |  |  |  |  |

**6. Social Science Citation Index**Interface/URL: Web of Science
Database coverage dates: 1975-present
Search date: 7 September 2020
Number of results retrieved: 51
Search strategy:

| # 7 | 51 | #5 NOT #6  *Indexes=SSCI Timespan=All years* |  |
| --- | --- | --- | --- |
| 6 | 60,914 | TI=("rat" or "rats" or "rodent" or "rodents" or "mouse" or "mice" or "murine" or "hamster" or "hamsters" or "gerbil" or "gerbils" or "animal" or "animals" or "dogs" or "dog" or "canine" or "pig" or "pigs" or "piglet" or "piglets" or "cat" or "cats" or "bovine" or "cow" or "cows" or "cattle" or "sheep" or "ewe" or "ewes" or "horse" or "horses" or "equine" or "ovine" or ”swine" or "porcine" or "monkey" or "monkeys" or "primate" or "primates" or "rhesus macaque" or "rhesus macaques" or "rabbit" or "rabbits") NOT TS=(human* or "women" or "woman" or "man" or "men" or "child" or "children" or adolescen* or teenager* or "people" or "boy" or "boys" or "girl" or "girls")  *Indexes=SSCI Timespan=All years* |  |
| # 5 | 54 | #3 AND #4  *Indexes=SSCI Timespan=All years* |  |
| # 4 | 394,093 | TS=(trial* OR random* OR "clinical" NEAR/3 stud* OR "controlled" NEAR/3 stud*)  *Indexes=SSCI Timespan=All years* |  |
| # 3 | 257 | #1 AND #2  *Indexes=SSCI Timespan=All years* |  |
| # 2 | 6,084 | TS=("calcium")  *Indexes=SSCI Timespan=All years* |  |
| # 1 | 85,889 | TS=(pregnan* OR gestation* OR antenatal* OR prenatal* OR postconception*) OR TS=(ante NEAR/3 natal* OR pre NEAR/3 natal* OR post NEAR/3 conception*)  *Indexes=SSCI Timespan=All years* |  |

**7. Conference Proceedings Citation Index – Science**Interface/URL: Web of Science
Database coverage dates: 1990-present
Search date: 7 September 2020
Number of results retrieved: 72
Search strategy:

| # 7 | 72 | #5 NOT #6  *Indexes=CPCI-S Timespan=All years* |
| --- | --- | --- |
| # 6 | 264,167 | TI=("rat" or "rats" or "rodent" or "rodents" or "mouse" or "mice" or "murine" or "hamster" or "hamsters" or "gerbil" or "gerbils" or "animal" or "animals" or "dogs" or "dog" or "canine" or "pig" or "pigs" or "piglet" or "piglets" or "cat" or "cats" or "bovine" or "cow" or "cows" or "cattle" or "sheep" or "ewe" or "ewes" or "horse" or "horses" or "equine" or "ovine" or ”swine" or "porcine" or "monkey" or "monkeys" or "primate" or "primates" or "rhesus macaque" or "rhesus macaques" or "rabbit" or "rabbits") NOT TS=(human* or "women" or "woman" or "man" or "men" or "child" or "children" or adolescen* or teenager* or "people" or "boy" or "boys" or "girl" or "girls")  *Indexes=CPCI-S Timespan=All years* |
| # 5 | 75 | #3 AND #4  *Indexes=CPCI-S Timespan=All years* |
| # 4 | 393,774 | TS=(trial* OR random* OR "clinical" NEAR/3 stud* OR "controlled" NEAR/3 stud*)  *Indexes=CPCI-S Timespan=All years* |
| # 3 | 544 | #1 AND #2  *Indexes=CPCI-S Timespan=All years* |
| # 2 | 52,403 | TS=("calcium")  *Indexes=CPCI-S Timespan=All years* |
| # 1 | 59,220 | TS=(pregnan* OR gestation* OR antenatal* OR prenatal* OR postconception*) OR TS=(ante NEAR/3 natal* OR pre NEAR/3 natal* OR post NEAR/3 conception*)  *Indexes=CPCI-S Timespan=All years* |

**8. Conference Proceedings Citation Index- Social Science & Humanities**Interface/URL: Web of Science
Database coverage dates: 1990-present
Search date: 7 September 2020
Number of results retrieved: no results retrieved
Search strategy:

| # 7 | 0 | #5 NOT #6  *Indexes=CPCI-SSH Timespan=All years* |
| --- | --- | --- |
| # 6 | 2,163 | TI=("rat" or "rats" or "rodent" or "rodents" or "mouse" or "mice" or "murine" or "hamster" or "hamsters" or "gerbil" or "gerbils" or "animal" or "animals" or "dogs" or "dog" or "canine" or "pig" or "pigs" or "piglet" or "piglets" or "cat" or "cats" or "bovine" or "cow" or "cows" or "cattle" or "sheep" or "ewe" or "ewes" or "horse" or "horses" or "equine" or "ovine" or ”swine" or "porcine" or "monkey" or "monkeys" or "primate" or "primates" or "rhesus macaque" or "rhesus macaques" or "rabbit" or "rabbits") NOT TS=(human* or "women" or "woman" or "man" or "men" or "child" or "children" or adolescen* or teenager* or "people" or "boy" or "boys" or "girl" or "girls")  *Indexes=CPCI-SSH Timespan=All years* |
| # 5 | 0 | #3 AND #4  *Indexes=CPCI-SSH Timespan=All years* |
| # 4 | 20,239 | TS=(trial* OR random* OR "clinical" NEAR/3 stud* OR "controlled" NEAR/3 stud*)  *Indexes=CPCI-SSH Timespan=All years* |
| # 3 | 5 | #1 AND #2  *Indexes=CPCI-SSH Timespan=All years* |
| # 2 | 411 | TS=("calcium")  *Indexes=CPCI-SSH Timespan=All years* |
| # 1 | 1,927 | TS=(pregnan* OR gestation* OR antenatal* OR prenatal* OR postconception*) OR TS=(ante NEAR/3 natal* OR pre NEAR/3 natal* OR post NEAR/3 conception*)  *Indexes=CPCI-SSH Timespan=All years* |

**9. ClinicalTrials.gov**Interface/URL: https://www.clinicaltrials.gov/
Database coverage dates: from inception to date
Search date: 7 September 2020
Number of results retrieved:
Search strategy:

Search terms were entered into the default search box on the initial screen.

Status: All studies

Condition or disease
pregnancy OR pregnant OR gestation OR antenatal OR prenatal OR postconception OR ante-natal OR pre-natal OR post-conception

Other terms
calcium

**10. WHO International Clinical Trials Platform**Interface/URL: https://apps.who.int/trialsearch/
Database coverage dates: from inception to date
Search date: 7 September 2020
Number of results retrieved: 110
Search strategy:

pregnancy AND calcium OR pregnant AND calcium OR gestation* AND calcium OR antenatal* AND calcium OR prenatal* AND calcium OR postconception* AND calcium OR ante-natal* AND calcium OR pre-natal* AND calcium OR post-conception* AND calcium

**11. PROSPERO -** **International prospective register of systematic reviews**Interface/URL: https://www.crd.york.ac.uk/prospero/
Database coverage dates: from inception to date
Search date: 7 September 2020
Number of results retrieved: 77
Search strategy:

Advanced search screen available at https://www.crd.york.ac.uk/prospero/#searchadvanced was used to run the search.

#1 MeSH DESCRIPTOR Pregnancy EXPLODE ALL TREES 3271

#2 MeSH DESCRIPTOR Pregnant women 111

#3 gestation* or antenatal* or prenatal* or postconception* or ante-natal* or pre-natal* or post-conception* 4631

#4 #1 OR #2 OR #3 5893

#5 MeSH DESCRIPTOR calcium 31

#6 MeSH DESCRIPTOR calcium, dietary 36

#7 calcium 1045

#8 #5 OR #6 OR #7 1049

#9 #4 AND #8 77
